# Fatty acids composition and lipolysis of Parmigiano Reggiano PDO cheese: effect of the milk cooling temperature at the farm

**DOI:** 10.5713/ab.22.0080

**Published:** 2022-06-30

**Authors:** Piero Franceschi, Paolo Formaggioni, Milena Brasca, Giuseppe Natrella, Michele Faccia, Massimo Malacarne, Andrea Summer

**Affiliations:** 1 Department of Veterinary Science, University of Parma, Via del Taglio 10, I-43126, Parma, Italy; 2 Institute of Sciences of Food Production, National Research Council, Via Celoria 2, IT-20133 Milano, Italy; 3 Department of Soil, Plant and Food Sciences, University of Bari, Via Amendola 165/A, 70125 Bari, Italy

**Keywords:** Cheese Fat Composition, Cheese Lipolysis, Fatty Acids, Milk Cooling, Parmigiano Reggiano

## Abstract

**Objective:**

The aim was to study the influence of cooling milk at 9°C at the farm versus keeping it at 20°C on Parmigiano Reggiano cheese lipolysis.

**Methods:**

A total of six cheesemaking trials (3 in winter and 3 in summer) were performed. In each trial, milk was divided continuously into two identical aliquots, one of which was kept at 9°C (MC9) and the other at 20°C (MC20). For each trial and milk temperature, vat milk (V-milk) and the resulting 21 month ripened cheese were analysed.

**Results:**

Fat and dry matter and fat/casein ratio were lower in MC9 V-milk (p≤0.05) than in MC20. Total bacteria, mesophilic lactic acid and psychrotrophic and lipolytic bacteria showed significant differences (p≤0.05) between the two V-milks. Regarding cheese, fat content resulted lower and crude protein higher (p≤0.05) both in outer (OZ) and in inner zone (IZ) of the MC9 cheese wheels. Concerning total fatty acids, the MC9 OZ had a lower concentration of butyric, capric (p≤0.05) and medium chain fatty acids (p≤0.05), while the MC9 IZ had lower content of butyric (p≤0.05), caproic (p≤0.01) and short chain fatty acids (p≤0.05). The levels of short chain and medium chain free fatty acids (p≤0.05) were lower and that of long chain fatty acids (p≤0.05) was higher in MC9 OZ cheese. The principal component analysis of total and free fatty acids resulted in a clear separation among samples by seasons, whereas slight differences were observed between the two different milk temperatures.

**Conclusion:**

Storing milk at 9°C at the herd affects the chemical composition of Parmigiano Reggiano, with repercussion on lipolysis. However, the changes are not very relevant, and since the cheese can present a high variability among the different cheese factories, such changes should be considered within the “normal variations” of Parmigiano Reggiano chemical characteristics.

## INTRODUCTION

Parmigiano Reggiano is a Protected Designation of Origin (PDO) cheese characterised by a long ripening time (from 12 to over 24 month), an average protein content of about 33% and a fat content ranging from 25.5% to 31.4% [1]. Like all PDO products, its production process is strictly regulated by an official manufacturing protocol [2]. As the protocol imposes that the cheese must be manufactured from raw milk, the chemical and microbiological characteristics of the raw matter strongly affect all phases of the transformation process [3,4], with repercussion on both cheese yield [5,6] and quality [1].

The manufacturing protocol not only imposes the rules for cheesemaking, but also regulates the production and management of the milk at the farm. In fact, strict rules are defined for the milking operations, the condition of milk keeping at the farm and the delivery of milk to the cheese factory. Regarding the two latter aspects, cows must be milked two times a day and the interval between start of the milking procedure and milk delivery to the cheese factory must be shorter than 7 hours [2]. In this period, the milk must be kept at the farm and transported to the cheese factory at a temperature not below of 18°C [2].

Such a temperature limit is critical, since it is known that adopting a lower cooling temperature better preserves milk from spoiling by reducing the growth of spoilage bacteria and slowing down the activity of proteolytic and lipolytic enzymes deriving from leukocytes [7–9]. For this reason, the milk destined to the production of hard cheeses is commonly kept at lower temperatures, as happens, as an example, in Grana Padano cheese manufacturing, in which the milk can be cooled down to 9°C.

On the other hand, lowering the temperature of milk keeping at the farm, might cause modifications in the chemical and microbiological characteristics [10,11] with consequent negative impact on the cheese ripening process [1]. Two important modifications are the decrease of coagulable casein [11] and the changes of the milk microbiological characteristics [10]. The former can lead to a cheese yield reduction [5,12], the latter can affect the cheese ripening process [1,13], including lipolysis [3,13].

It is known that lipolysis in cheese is caused by lipolytic enzymes both endogenous and coming from the lysis of lactic acid bacteria [13], lipolytic and psychrotrophic bacteria [14]. These enzymes are both lipases and esterases that catalyse the hydrolysis of the ester bond between glycerol and fatty acids resulting in the production of free fatty acids (FFA), and mono or diacylglycerides [15]. In cheese, FFA with short- and medium-chains directly contribute to the cheese flavour [13], and can also act as precursors of many flavour compound, such as esters, alkanes, methyl ketones, lactones and secondary alcohols [13].

Reducing the storage temperature of milk to 9°C helps to contain Gram negative psychrotrophic bacteria harbouring spoilage features and can also reduce the production of extracellular proteases, lecithinases and lipases [16].

Since lipolysis produces FFA that contribute to the typical cheese flavour, keeping the milk at 9°C at the farm should influence the sensory quality of the ripened cheese.

The aim of this research was to study the effect of cooling the milk at 9°C at the farm versus keeping it at 20°C on the lipolysis process of Parmigiano Reggiano PDO cheese.

## MATERIALS AND METHODS

### Experimental design, sampling procedure and classification of cheese batches

A total of six cheesemaking trials (3 in winter and 3 in summer) were performed using the milk deriving from a farm raising Italian Friesian cows. In each trial, during both evening and morning milking, the raw matter coming from the milking parlor was divided continuously into two identical aliquots that were placed in two different cooling tanks. In the first one, milk was kept at 9°C (MC9 milk) and in the second one it was kept at 20°C (MC20 milk). At the end of the milking procedure, lasting about three hours, both MC9-milk and MC20-milk were delivered separately to the cheese factory and submitted to the cheesemaking process. During the transport, lasting about one hour, MC9 and MC20 milks were kept at the same temperature than at the farm.

As soon as they arrived at the cheese factory, the milk was subjected in parallel to cheesemaking according to the PDO official manufacturing protocol [2] of Parmigiano Reggiano, except for the milk cooling temperature used for MC9 milk.

For each cheesemaking trial, a sample of vat milk (V-milk) was collected and, after 21 months of ripening, a MC9 and a MC20 cheese wheel was taken, for the analyses.

**Figure 1:**
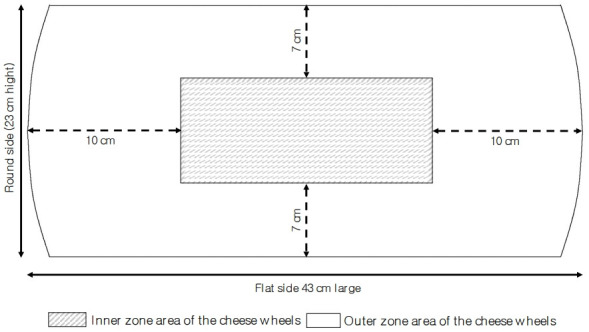
Scheme of sampling from the vertical section of the Parmigiano Reggiano cheese wheel.

Sampling of the cheese was carried out as described by Malacarne et al [17]. In short, a medial section was taken from the cheese wheels and this medial section was cut from the round side to the centre to obtain a vertical section 4 cm thick from which, after removal of the rind (5 to 7 mm), a sample representative of the outer zone and a sample representative of the inner zone were collected (Figure 1). The obtained samples were kept a 4°C and analysed within 2 days.

Such a sampling procedure is necessary since, after brine salting, sodium chloride is concentrated in the outer zone of the cheese wheel and takes about 12 months to diffuse in the inner part. This phenomenon determines different environmental conditions in terms of salt concentration and water activity in the different zones of the cheese wheels [17], which affect the microbial and the enzymatic activities during ripening [17].

### Cheesemaking process

The whole evening milk was collected at the farm directly from the cooling tanks and, by a tank truck, it was delivered to the cheese factory, where it was placed into a creaming tank overnight. During this period, natural creaming took place and, the morning after, the partially skimmed milk was extracted and transferred into the cheesemaking vat and added with the full cream morning milk just arrived from the farm. The result of this commingling is named “vat milk” and contains approximately 2.6% fat and a casein to fat ratio of about 1.1. The cheesemaking process started with the addition of a natural whey starter culture (about 3 liters per 100 kg of milk), prepared by overnight fermentation of the whey from the cheesemaking of the previous day. Successively, the vat milk was clotted with 2.5 g/100 kg milk of powdered calf rennet with a strength of 1:120,000 and with 98% of chymosin and 2% of pepsin (Caglio Bellucci Srl, Modena, Italy). Coagulation occurred in about 12 minutes and, after 2 minutes of firming, the curd was cut into small grains (around 5 millimeters of size). Then, the curd was cooked by increasing the temperature up to 55°C. During this phase, the mass was continually stirred to ensure uniform heating and to avoid settling of the grains. At the end of the cooking phase the cheese grains were left to settle on the bottom of the vat for about one hour. Finally, the curd mass was extracted, divided into two parts to form two twin cheese wheels that remained in the moulds for about two days, to promote syneresis and a correct acidification. Then, the cheese wheels were salted for 18 days in saturated brine (salt concentration of 357 g/L), and transferred into the ripening room, where they remained for 21 months at 18°C and at 80% of relative humidity.

### Analytical methods

The total and non-casein nitrogen fractions were determined by Kjeldahl method on each milk sample and on acid whey at pH 4.6, respectively. From these nitrogen fractions, crude protein, casein, and casein number were calculated according to Malacarne et al [18].

Moreover, the lactose and fat contents were assessed on V-milk samples by MilkoScan (Foss Electric, Hillerød, Denmark) and the fat to casein ratio was calculated.

Dry matter content on V-milk was obtained after oven-drying at 102°C and the solid-not-fat value was calculated.

The V-milk total bacterial and somatic cells counts were determined using the flow cytometry method with BactoScan FC (Foss Electric, Denmark) and fluoro-opto-electronic method with Fossomatic (Foss Electric, Denmark), respectively.

For microbiological analyses, serial decimal dilutions were plated on the following media: De Man, Rogosa, Sharpe Agar (MRS; Oxoid, Thermo Fisher Scientific, Basingstoke, UK) at 21°C for 5 days of incubation to count mesophilic lactic acid bacteria; Plate Count Agar (PCA; Oxoid, Thermo Fisher Scientific, UK) at 6.5°C for 10 days of incubation for the enumeration of psychrotrophic bacteria; Tributyrin agar (Oxoid, Thermo Fisher Scientific, UK) at 30°C for 7 days of incubation for lipolytic bacteria [19]. The counts were expressed as logarithms of colony-forming units per mL (Log CFU/mL).

As to cheese, the total nitrogen (TN) content was determined by Kjeldahl method, from which the value of crude protein (TN×6.38/1,000) was calculated [20]; the fat content was determined by the volumetric method of Gerber; the sodium chloride content was assessed by titration with AgNO_3_. Dry matter and ash were determined by oven drying of cheese at 102°C and by muffle calcination at 530°C, respectively.

Total fatty acids (TFA) were determined according to the Association of Official Analytical Chemists (AOAC) standard methods [21]. Briefly, lipids were extracted from approximately 1 g of grated cheese, after the addition of sodium sulphate and of 2.5 M sulphuric acid (Carlo Erba, Milan, Italy), using a mixture of ether–heptane (rate in volume 1:1). After the extraction, lipids were derivatised and their content was determined by capillary chromatography with a Carlo Erba GC 6000 Vega Series gas chromatograph (Carlo Erba, Italy). The chromatography was equipped with a fused silica capillary column coated with Polyethylene Glycol Supelco, SP TM 2330 (Sigma-Aldrich Corporation, Saint Louis, MO, USA). Each fatty acid ester was then identified by comparing the retention times with those of a standard solution.

Free fatty acids were determined according to the method of De Jong and Badings [22]. In brief, lipids were extracted from ground cheese as previously described for TFA and, to isolate FFA, the lipid extract was applied to a specific amino-propyl column (Carlo Erba, Italy). The non-adsorbed FFA were separated and determined yet by capillary chromatography also with a Carlo Erba GC 6000 Vega Series gas chromatographer (Carlo Erba, Italy). The chromatograph, this time, was equipped with a fused silica capillary column, coated with Polyethylene Glycol, model AT 1000 (Alltec Associates Inc., Deerfiled, IL, USA). As for TFA, also FFA were identified by comparing the retention times with those of a standard solution.

For both total and FFA, short chain fatty acids (SCFA) were calculated by the sum of the compounds from C4 to C8; medium chain fatty acids (MCFA) by the sum of the compounds from C10 to C14, and long chain fatty acids (LCFA) by the sum of the compounds from C16 to C18, according to Malacarne et al [17].

Moreover, from the values of the single fatty acids, the contents of saturated and unsaturated fatty acids were calculated and, among the unsaturated fatty acids, the content of monounsaturated and polyunsaturated fatty acids were calculated.

### Statistical analysis

Data collected were submitted to analysis of variance, using the general linear model procedure of software SPSS version 27 (IBM SPSS Statistics version 27, Armonk, New York, USA), and the least square mean values were calculated according to the following univariate model:


$${\text{Y}}_{\text{ijk}}=\mu +{\text{C}}_{\text{i}}+{\text{T}}_{\text{j}}+{ɛ}_{\text{ijk}}$$

where: Y_ijk_ = dependent variable; μ = overall mean; C_i_ = effect of the temperature, 9°C or 20°C (i = 1, 2); T_j_ = effect of trial (j = 1,...6); ɛ_ijk_ = residual error.

The significance of the differences between cheese produced with milk kept at the farm at 9°C and cheese produced with milk kept at the farm at 20°C was tested by means of Bonferroni method.

Furthermore, data were submitted to the principal component analysis (PCA) considering the two different milk temperature at the herd and the zone of sampling of the cheese wheels and considering the two different milk temperature at the herd and the season of the trials. Xlstat-sensory software (Addinsoft, Paris, France) was used.

## RESULTS AND DISCUSSION

The chemical composition and the microbiological characteristics of the V-milk obtained from milk kept at 9°C and 20°C are shown in Table 1. The fat and dry matter contents and the fat to casein ratio value resulted lower in V-milk obtained from milk kept at 9°C compared to the one kept at 20°C (p≤0.05). This is due to the higher creaming capacity and the consequent lower fat content of the partially skimmed milk obtained from the first milk compared to the second one. However, although the difference between the mean values was statistically significant, both the mean values of V-milk fat to casein ratio fell in the range 1–1.1, which is considered optimal to produce Parmigiano Reggiano [23].

Total viable mesophilic lactic acid bacteria, as well as psychrotrophic and lipolytic bacteria counts, showed significant differences (p≤0.05) between V-milk obtained from the milk kept at 9°C with respect to that at 20°C. These differences confirmed the slower growth of all these bacteria at 9°C, as previously reported by Malacarne et al [24], who found a significant difference of the total bacterial count value between the milk kept at 8°C to 10°C for 12 hours and the milk kept at 13°C to 15°C (67,600 to 100,000 CFU/mL vs 104,700 to 218,800 CFU/mL, respectively).

### Effect of the of milk storage temperature at the farm on cheese fatty acid content

The chemical composition and total fatty acid contents of the cheese samples are shown in Tables 2 and 3. The fat content both in the outer and in the inner zones of cheese from MC9 milk was lower and that of crude protein higher (p≤0.05) than in the cheese from MC20 milk. These results derived from the different fat content (2.43 vs 2.53 g/100 g: p≤0.05, respectively) and fat to casein ratio (1.01 vs 1.05: p≤0.05, respectively) in the corresponding vat milks. Despite of the statistically significance, it is worth noting that the differences in the cheese composition were very small: the value of crude protein was in accordance with that reported by Mammi et al [25] whose research aimed to evaluate the effect of a mass treatment of dry cows with Monensin on Parmigiano Reggiano cheese production, reported the average protein value of 44.61 g/100 g of dry matter in the cheese ripened for 18 months. On the other hand, the same study reported an average value for fat content of 48.86 g/100 g of dry matter. More recently, D’Incecco et al [26], in an investigation on the characteristics of Parmigiano Reggiano cheese during a ripening period up to 50 months, reported similar average values of fat content for cheese 18 months ripened (42.08 g/100 g of dry matter) and for cheese 24 months ripened (46.15 g/100 g of dry matter). Overall, the fat values observed in this study should be considered within the normal range of variation in an artisanal product as is Parmigiano Reggiano, in accordance with Careri et al [27], who reported a high variability (from 41.41 to 44.54 g/100 g of dry matter) of the fat content in batches of Parmigiano Reggiano ripened for 24 months produced in different cheese factories.

Regarding the total fatty acid composition, the outer zone of the cheese wheels produced with MC9 milk presented a lower content of butyric and capric acids (p≤0.05) and of MCFA (p≤0.05) compared to the outer zone of the cheese wheels produced with MC20 milk. The differences were not the same in the inner zones, since MC9 cheese was characterised by lower contents of butyric (p≤0.05) and caproic (p≤ 0.01) acids and of SCFA (p≤0.05) with respect to the MC20 cheese. These differences in the TFA composition could be connected with the higher creaming capacity of the milk kept at 9°C than that at 20°C, in accordance with Malacarne et al [24], who observed a higher creaming capacity of milk cooled at 8°C to 10°C compared to that maintained at 13°C to 15°C (42.0% vs 38.5%). In the present research, the higher creaming capacity of MC9 milk respect to the MC20 was much more evident (53.44% vs 47.73%: p≤0.05, data not shown) and was probably due to a more pronounced breaking of the fat globules at lower temperature, with consequent leaking of triglycerides [28]. This phenomenon favoured their aggregation in large clusters that tend to surface more effectively [28] and might have favoured the separation of the less dense globules, containing higher levels of short-chain fatty acids such as butyric and caproic. Despite these differences, the total fatty acid profile of both cheeses was consistent with the literature data (Mammi et al [25]).

However, although significant, the entity of the differences between the mean values of free butyric and caproic acids are small and probably not able to affect the flavors of the cheeses. This hypothesis is supported by the observation that the values of both FFA are slightly lower than those reported by Malacarne et al [17].

### Effect of milk storage temperature at the farm on lipolysis of the cheese

Tables 4 and 5 show the FFA contents of the cheese samples. According to the literature, the total FFA content is expected to be higher in outer than in inner zone of the cheese wheel [17]. The values in the inner zone found in the present study were consistent with those reported by many authors [17,29]. Malacarne et al [17] reported an average total FFA content of 4,636.90 g/kg in 18 months ripened cheese and of 7,485.68 g/kg in 24 months ripened cheese.

On the other hand, the FFA content in the outer zone was lower than that reported in the above studies. Malacarne et al [17] reported an average value of total FFA of 7,203.40 and 10,726.82 g/kg in cheeses ripened for 18 and 24 months, respectively.

As to the single FFA, the outer zone of the cheese wheels produced with MC9 milk, compared to those produced with MC20 milk, presented lower contents of short chain and MCFA (p≤0.05) and, consequently, higher level of LCFA (p≤0.05). These differences could be related to the lower content of lipolytic (2.94 vs 3.46 Log CFU/mL, p≤0.05; respectively) and psychrotrophic (2.73 vs 3.12 Log CFU/mL, p≤0.05; respectively) bacteria in vat milk maintained at 9°C, respect to that maintained at 20°C. In fact, these bacterial groups can release carboxylesterases that hydrolyse the ester bond of acylglycerols [30,31], resulting in strong formation of short- and medium- chain FFA with respect to the long-chain FFA [31,32].

### Result of the principal component analysis of the total and free fatty acids

**Figure 2:**
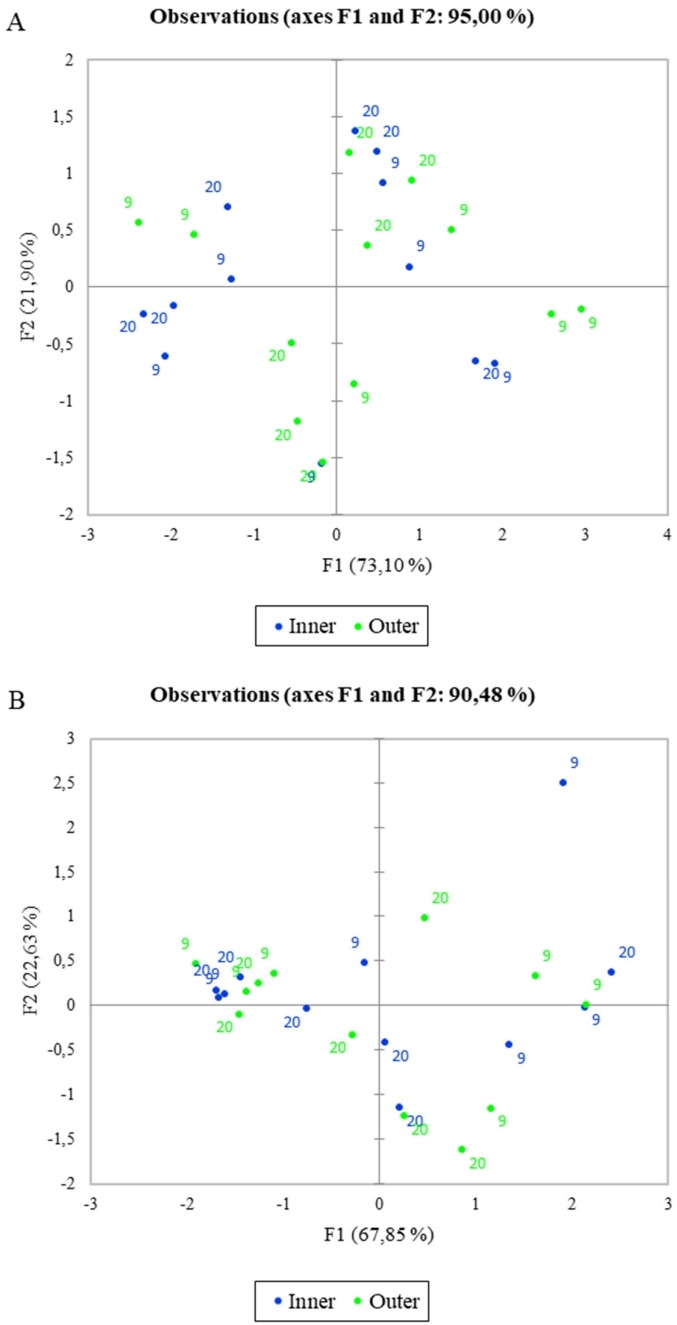
Principal component analysis of the total (A, g/100 g of fatty acids) and free (B, mg/100 g of fat) fatty acids considering the different milk temperature and the inner/outer zone of the cheese wheel.

**Figure 3:**
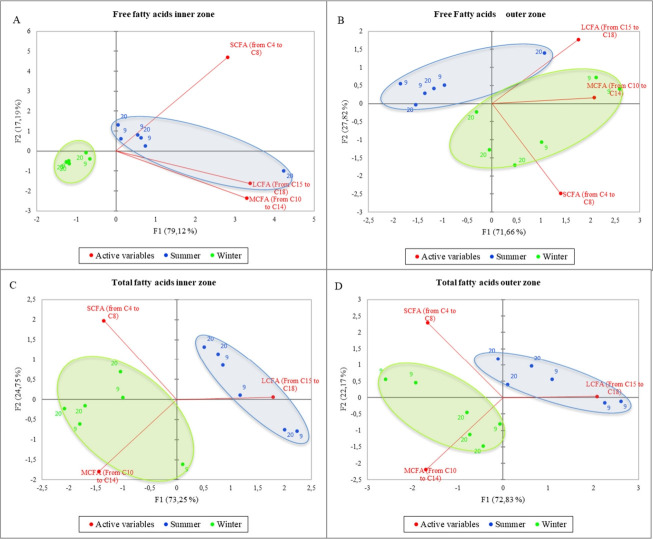
Principal component analysis of the free (A,B) and total (C,D) fatty acids considering the different milk temperature and the sampling seasons.

Figure 2 shows the PCA of the total (A) and free (B) short, medium, and long fatty acids considering the different milk temperature and the inner/outer zone of the cheese wheels. The variance is well explained in both graphs (95% and 90.48%, respectively), and PC1 is the axis with higher percentage of variance explanation. Nevertheless, samples belonging to the same category are not well grouped, in fact, it is possible to observe a well-mixed spreading of all samples within the PCA space, meaning that the differences among samples are poor or that the differences previously found are not only strictly related to the milk storage temperature. To better understand the information behind these results, a deeper elaboration was done by including the season of sampling as a further variable in the PCA computation process. The resulting output are shown in the Figure 3. More in detail, the figure reports the PCA of the free (A,B) and total (C,D) fatty acids, considering both the milk temperature and the sampling seasons. Also in this case, the variability explained is very high in all graphs (ranging from 96.31% to 99.48%), but a new insight emerged: in fact, a clear separation among samples by seasons was obtained, whereas still little differences were observed when considering the milk temperature. The PCA results confirm that the milk temperature did not affect the lipolysis process, differently from the season. In Figure 3A, the winter samples laid on the negative side of PC1 and in a small portion of the plot, whereas most of the summer ones were gathered near the axes origin on the positive side of PC1 and only one sample was far from the others, showing a higher correlation with LCFA and MCFA. In general, the graph suggests a higher content of FFA in the inner zone of the cheese made in summer than in the cold season. In Figure 3B, representing the FFA content in the outer zone of the wheel, unlike the previous figure, the summer samples laid on the negative side of PC1 having less correlation with FFA content, except for one sample which resulted strongly related to LCFA as before. On the other hand, winter samples were placed mostly on the positive side of PC1, having higher correlation with MCFA and SCFA. Figure 3C and 3D showed similar active variables orientation, being SCFA and MCFA oriented towards the negative side of PC1 and LCFA to the positive side of the same axis. The samples from milk stored at different temperature still “mixed together”, and once again the samples were only discriminated by season: the winter ones laid on the negative side of PC1, being more related to MCFA and SCFA, both in the inner and outer zone; on the contrary, the summer samples laid on the positive side of PC1 (except for one sample in Figure 3D), being related to a higher LCFA content. In general, among all figures, PC1 is the axis that mostly discriminate samples, splitting the summer samples on the positive side, and winter sample on the negative side of the plot, except for Figure 3B, in which the two groups result to be inverted. Free fatty acids of the summer samples were higher in the inner zone (3A) of the cheese wheel and lower in the outer zone (3B). On the other hand, the winter samples result poorer in FFA in the inner zone during winter, and richer in the outer zone. Considering the total fatty acids (3C, D), the summer samples, both in the inner and in the outer zone, were well correlated to LCFA, whereas the winter samples, in both graphs, were more related to MCFA and SCFA.

As reported in several researches, chemical composition, physico-chemical and microbial characteristics of milk for Parmigiano Reggiano significantly vary among seasons, with repercussion on cheese yield and quality. For example, the temperature and humidity values that characterize the summer season in the Parmigiano Reggiano geographical area can induce heat stress in cows [33]. This leads to a reduction in milk yield and fat and protein contents, as well as a worsening of its rennet coagulation and cheese yielding ability [4,33]. Moreover, the change in the photoperiod that takes place during the year (daylight maximum in summer and minimum in winter) can also affect yield and quality of milk [33].

Indeed, Pacheco-Pappenheim et al [34], in a study aimed to investigate the effect of seasonal variation on the changes of fatty acids and in bovine milk fat, showed a higher LCFA content in the milk produced in the summer season respect to the one produced in the winter one.

## CONCLUSION

In conclusion, storing the milk at 9°C at the farm can affect the chemical composition of Parmigiano Reggiano cheese, mainly for the fat content and fatty acid profile, with significant repercussion on cheese lipolysis during the ripening phase. However, the size of the differences appears rather small, and since this cheese is characterised by a high variability among different cheese factories, the differences observed in the fatty acid profile and lipolysis should be considered within the “normal variations” of the chemical characteristics. The impact of the season seems to be much more relevant than the temperature of milk storage and might be the most important source of variation of the chemical characteristics of Parmigiano Reggiano.

## Figures and Tables

**Table 1:** Chemical composition, physicochemical properties parameters and microbiological characteristics of the vat milk obtained from the herd milk stored at the farm at 9°C (MC9) or 20°C (MC20) (least square mean values)

Variable		MC9	MC20	SE	p-value
	
		N^1)^ = 6	n^1)^ = 6		
Lactose	g/100 g	4.88	4.89	0.01	NS
Fat	g/100 g	2.43	2.53	0.03	^*^
Crude protein	g/100 g	3.09	3.11	0.02	NS
Casein	g/100 g	2.40	2.41	0.02	NS
Casein number	%	77.40	77.53	0.13	NS
Dry matter	g/100 g	11.19	11.33	0.04	^*^
Matter not fat	g/100 g	8.76	8.80	0.02	NS
Fat to casein ratio	Value	1.01	1.05	0.01	^*^
Total bacterial count	Log CFU/mL	4.45	4.85	0.11	^*^
Mesophilic lactic acid bacteria	Log CFU/mL	3.02	3.37	0.09	^*^
Psychrotrophic bacteria	Log CFU/mL	2.73	3.12	0.18	^*^
Lipolytic bacteria	Log CFU/mL	2.94	3.46	0.15	^*^
Somatic cells count	Log cells/mL	5.09	5.14	0.02	NS

SE, standard error; CFU, colony-forming units.

1) Number of samples.

NS (not significant) p>0.05,

* p≤0.05

**Table 2:** Chemical composition and total fatty acid content of the outer zone of cheese wheels produced by milk kept at the farm at 9°C (MC9) or 20°C (MC20) (least square mean values)

Variable		MC9	MC20	SE	p-value
	
		n^1)^ = 6	n^1)^ = 6		
Dry matter	g/100 g	70.28	69.97	0.11	NS
Fat	g/100 g of dry matter	42.57	43.45	0.34	^*^
Crude protein	g/100 g of dry matter	46.86	45.64	0.45	^*^
Ash	g/100 g of dry matter	6.18	5.99	0.06	NS
Salt (NaCl)	g/100 g of dry matter	1.91	1.92	0.07	NS
Butyric (C4)	mg/100 g	563.64	620.59	91.45	^*^
Caproic (C6)	mg/100 g	343.68	347.63	29.84	NS
Caprylic (C8)	mg/100 g	248.60	215.80	27.27	NS
Capric (C10)	mg/100 g	586.06	612.61	9.07	^*^
Lauric (C12)	mg/100 g	744.63	767.05	11.54	NS
Mirystic (C14)	mg/100 g	2,228.54	2,248.75	55.55	NS
Myristoleic (C14:1)	mg/100 g	256.16	262.28	9.52	NS
Pentadecanoic (C15)	mg/100 g	346.26	314.17	15.66	NS
Palmitic (C16)	mg/100 g	8,082.16	7,935.43	239.34	NS
Palmitoleic (C16:1)	mg/100 g	600.27	568.60	17.31	NS
Margaric (C17)	mg/100 g	240.09	214.71	9.01	NS
Stearic (C18)	mg/100 g	3,010.67	2,900.79	88.91	NS
Oleic (C18:1)	mg/100 g	7,193.32	7,175.86	166.86	NS
Linoleic (C18:2)	mg/100 g	884.50	878.59	18.76	NS
Linolenic (C18:3)	mg/100 g	140.89	140.35	23.28	NS
SCFA (from C4 to C8)	mg/100 g	1,155.92	1,184.01	130.58	NS
MCFA (From C10 to C14)	mg/100 g	3,815.40	3,890.68	56.34	^*^
LCFA (From C15 to C18)	mg/100 g	20,498.17	20,128.49	459.48	NS
Saturated fatty acids	mg/100 g	16,394.33	16,177.51	360.79	NS
Unsaturated fatty acids	mg/100 g	9,075.15	9,025.67	201.01	NS
Monounsaturated fatty acids	mg/100 g	8,049.76	8,006.74	147.64	NS
Polyunsaturated fatty acids	mg/100 g	1,025.39	1,018.93	27.18	NS
SCFA (from C4 to C8)	g/100 g of fatty acid	4.57	4.70	0.57	NS
MCFA (from C10 to C14)	g/100 g of fatty acid	14.98	15.42	0.12	^*^
LCFA (from C15 to C18)	g/100 g of fatty acid	80.46	79.88	0.61	NS
Saturated fatty acids	g/100 g of fatty acid	64.32	64.15	0.63	NS
Unsaturated fatty acids	g/100 g of fatty acid	35.68	35.85	0.63	NS
Monounsaturated fatty acids	g/100 g of fatty acid	31.65	31.79	0.55	NS
Polyunsaturated fatty acids	g/100 g of fatty acid	4.03	4.06	0.09	NS

SE, standard error; SCFA, short chain fatty acids; MCFA, medium chain fatty acids; LCFA, long chain fatty acids.

1) Number of samples.

NS (not significant) p>0.05,

* p≤0.05.

**Table 3:** Chemical composition and total fatty acid content of the inner zone of cheese wheels produced by milk kept at the farm at 9°C (MC9) or 20°C (MC20) (least square mean values)

Variable		9°C (MC9)	20°C (MC20)	SE	p-value
	
		n^1)^ = 6	n^1)^ = 6		
Dry matter	g/100 g	66.99	66.68	0.14	NS
Fat	g/100 g of dry matter	42.12	43.02	0.35	^*^
Crude protein	g/100 g of dry matter	47.16	46.22	0.36	^*^
Ash	g/100 g of dry matter	5.93	5.88	0.04	NS
Salt (NaCl)	g/100 g of dry matter	1.93	1.88	0.07	NS
Butyric (C4)	mg/100 g	575.82	735.13	40.58	^*^
Caproic (C6)	mg/100 g	333.11	401.69	9.11	^**^
Caprylic (C8)	mg/100 g	212.38	227.67	12.81	NS
Capric (C10)	mg/100 g	602.01	619.87	22.70	NS
Lauric (C12)	mg/100 g	744.19	763.21	18.25	NS
Mirystic (C14)	mg/100 g	2,165.33	2,240.74	33.31	NS
Myristoleic (C14:1)	mg/100 g	255.79	255.84	7.30	NS
Pentadecanoic (C15)	mg/100 g	354.83	321.84	27.71	NS
Palmitic (C16)	mg/100 g	7,638.93	7,885.07	154.02	NS
Palmitoleic (C16:1)	mg/100 g	547.92	563.76	24.36	NS
Margaric (C17)	mg/100 g	249.84	207.44	21.24	NS
Stearic (C18)	mg/100 g	2,847.16	2,851.87	65.18	NS
Oleic (C18:1)	mg/100 g	6,621.18	7,123.57	173.39	NS
Linoleic (C18:2)	mg/100 g	781.81	876.79	35.31	NS
Linolenic (C18:3)	mg/100 g	109.43	120.06	3.41	NS
SCFA (from C4 to C8)	mg/100 g	1,121.31	1,364.49	41.15	^*^
MCFA (From C10 to C14)	mg/100 g	3,767.32	3,879.66	76.12	NS
LCFA (From C15 to C18)	mg/100 g	19,178.10	19,950.04	379.06	NS
Saturated fatty acids	mg/100 g	15,723.61	16,254.17	289.02	NS
Unsaturated fatty acids	mg/100 g	8,384.12	8,940.02	219.35	NS
Monounsaturated fatty acids	mg/100 g	7,451.88	7,943.17	189.73	NS
Polyunsaturated fatty acids	mg/100 g	891.24	996.86	37.62	NS
SCFA (from C4 to C8)	g/100 g of fatty acid	4.62	5.44	0.21	^*^
MCFA (From C10 to C14)	g/100 g of fatty acid	15.60	15.39	0.08	NS
LCFA (From C15 to C18)	g/100 g of fatty acid	79.78	39.17	0.22	NS
Saturated fatty acids	g/100 g of fatty acid	65.13	64.52	0.53	NS
Unsaturated fatty acids	g/100 g of fatty acid	34.88	35.48	0.53	NS
Monounsaturated fatty acids	g/100 g of fatty acid	31.10	31.52	0.41	NS
Polyunsaturated fatty acids	g/100 g of fatty acid	3.78	3.96	0.14	NS

SE, standard error; SCFA, short chain fatty acids; MCFA, medium chain fatty acids; LCFA, long chain fatty acids.

1) Number of samples.

NS (not significant difference) p>0.05,

* p≤0.05,

** p≤0.01.

**Table 4:** Free fatty acid composition of the outer zone of cheese wheels produced by milk kept at the farm at 9°C (MC9) or 20°C (MC20) (least square mean values)

Variable		MC9	MC20	SE	p-value
	
		n^1)^ = 6	n^1)^ = 6		
Butyric (C4)	mg/100 g	20.20	20.04	1.13	NS
Caproic (C6)	mg/100 g	16.72	19.66	1.04	NS
Caprylic (C8)	mg/100 g	5.82	6.03	0.54	NS
Capric (C10)	mg/100 g	11.44	10.25	0.74	NS
Lauric (C12)	mg/100 g	12.73	9.58	2.20	NS
Mirystic (C14)	mg/100 g	64.83	58.37	11.36	NS
Paomitic (C16)	mg/100 g	176.45	143.46	33.04	NS
Palmitoleic (C16:1)	mg/100 g	27.15	29.36	5.95	NS
Stearic (C18)	mg/100 g	57.31	42.52	10.75	NS
Oleic (C18:1)	mg/100 g	133.66	86.79	29.35	NS
Linoleic (C18:2)	mg/100 g	26.83	18.76	6.70	NS
Total free fatty acid	mg/100 g	553.35	477,81	100.35	NS
SCFA (from C4 to C8)	g/100 free fatty acid	7.47	11.70	1.03	^*^
MCFA (From C10 to C14)	g/100 free fatty acid	15.87	18.16	0.41	^*^
LCFA (From C16 to C18)	g/100 free fatty acid	76.66	70.15	1.54	^*^
Butyric (C4)	mg/100 g of fat	68.74	74.91	3.75	NS
Caproic (C6)	mg/100 g of fat	56.22	63.67	3.08	NS
Caprylic (C8)	mg/100 g of fat	19.58	19.61	1.80	NS
Capric (C10)	mg/100 g of fat	38.56	33.29	2.66	NS
Lauric (C12)	mg/100 g of fat	42.92	31.57	7.57	NS
Mirystic (C14)	mg/100 g of fat	218.80	192.16	39.81	NS
Palmitic (C16)	mg/100 g of fat	596.14	472.11	115.40	NS
Palmitoleic (C16:1)	mg/100 g of fat	91.66	97.44	20.54	NS
Stearic (C18)	mg/100 g of fat	193.69	139.94	37.50	NS
Oleic (C18:1)	mg/100 g of fat	451.67	287.64	100.95	NS
Linoleic (C18:2)	mg/100 g of fat	90.55	62.48	22.85	NS
Total free fat acid	mg/100 g of fat	1,868.52	1,474.59	349.10	NS
SCFA (from C4 to C8)	mg/100 g of fat	144.53	158.18	8.37	NS
MCFA (From C10 to C14)	mg/100 g of fat	300.28	256.81	49.31	NS
LCFA (From C16 to C18)	mg/100 g of fat	1,423.71	1,059.60	294.40	NS

SE, standard error; SCFA, short chain fatty acids; MCFA, medium chain fatty acids; LCFA, long chain fatty acids.

1) Number of samples.

NS (not significant) p>0.05,

* p≤0.05.

**Table 5:** Free fatty acid composition of the inner zone of cheese wheels produced by milk kept at the farm at 9°C (MC9) or 20°C (MC20) (least square mean values)

Variable		MC9	MC20	SE	p-value
	
		n^1)^ = 6	n^1)^ = 6		
Butyric (C4)	mg/100 g	19.15	20.24	0.90	NS
Caproic (C6)	mg/100 g	14.64	16.49	0.61	NS
Caprylic (C8)	mg/100 g	5.38	5.50	0.25	NS
Capric (C10)	mg/100 g	10.59	12.87	1.35	NS
Lauric (C12)	mg/100 g	13.48	11.54	0.95	NS
Mirystic (C14)	mg/100 g	60.82	47.04	6.90	NS
Palmitic (C16)	mg/100 g	228.29	207.75	12.22	NS
Palmitoleic (C16:1)	mg/100 g	60.14	51.22	4.05	NS
Stearic (C18)	mg/100 g	60.39	62.03	3.69	NS
Oleic (C18:1)	mg/100 g	163.32	160.69	12.89	NS
Linoleic (C18:2)	mg/100 g	18.83	20.02	7.33	NS
Total free faty acid	mg/100	655.03	619.40	45.67	NS
SCFA (from C4 to C8)	g/100 free fatty acid	6.68	8.48	1.36	NS
MCFA (From C10 to C14)	g/100 free fatty acid	15.41	14.24	0.43	NS
LCFA (From C16 to C18)	g/100 free fatty acid	77.92	77.29	1.42	NS
Butyric (C4)	mg/100g of fat	68.48	71.27	3.34	NS
Caproic (C6)	mg/100g of fat	52.32	58.25	2.35	NS
Caprylic (C8)	mg/100g of fat	19.22	19.48	0.95	NS
Capric (C10)	mg/100g of fat	37.85	45.41	4.87	NS
Lauric (C12)	mg/100g of fat	48.15	40.57	3.25	NS
Mirystic (C14)	mg/100g of fat	217.03	164.33	24.05	NS
Palmitic (C16)	mg/100g of fat	816.08	732.25	52.98	NS
Palmitoleic (C16:1)	mg/100g of fat	215.14	181.95	14.26	NS
Stearic (C18)	mg/100g of fat	215.92	218.02	13.20	NS
Oleic (C18:1)	mg/100g of fat	583.60	567.54	45.44	NS
Linoleic (C18:2)	mg/100g of fat	67.14	84.64	26.15	NS
SCFA (from C4 to C8)	mg/100g of fat	140.01	149.00	6.50	NS
MCFA (From C10 to C14)	mg/100g of fat	303,03	250,31	28,93	NS
LCFA (From C16 to C18)	mg/100g of fat	1897.87	1784,40	140,11	NS
Total free fat acid	mg/100g of fat	2340.91	2183.70	160.88	NS

SE, standard error; SCFA, short chain fatty acids; MCFA, medium chain fatty acids; LCFA, long chain fatty acids.

1) Number of samples.

NS (not significant difference) p>0.05,

* p≤0.05.
